# Supplementary material for: Predicting Complete Cytoreduction with Preoperative [18F]FDG PET/CT in Patients with Ovarian Cancer: A Systematic Review and Meta-Analysis
Source: Diagnostics (Basel). 2024 Aug 10;14(16):1740. doi: 10.3390/diagnostics14161740 (PMC11353955; doi:10.3390/diagnostics14161740)
Supplement: Supplementary file 1 [file diagnostics-14-01740-s001.zip › diagnostics-3134698-supplementary.pdf]

Predicting complete cytoreduction with preoperative [18F]FDG PET/CT in patients with ovarian cancer: a systematic review and meta-analysis

Supplementary Materials

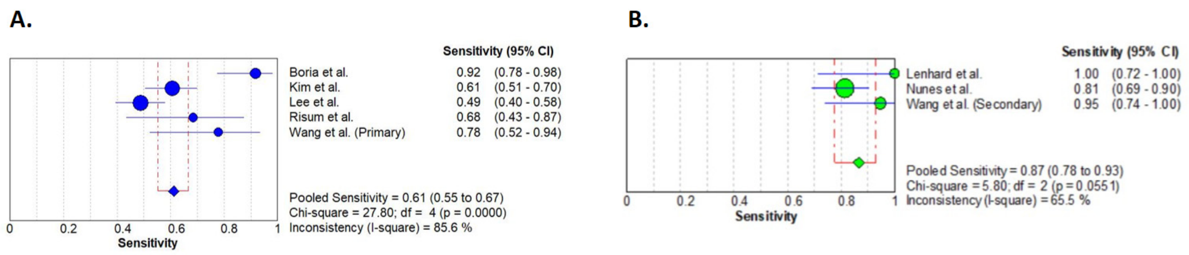

Figure S1. Forest plot analyses of sensitivities with Tsoi et al. excluded. Primary cytoreductive cases (**A**) separated from secondary cytoreductive cases (**B**). Results of the individual studies are represented by the circles. Circle sizes represent the weight of the studies. Pooled sensitivities are shown by the squares. Horizontal lines indicate the confidence intervals [21–28].

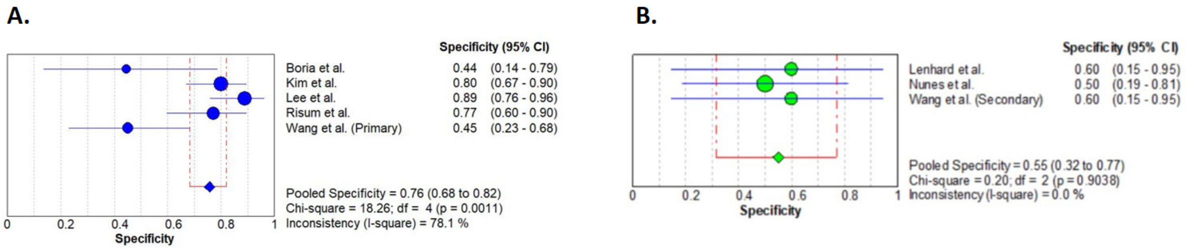

Figure S2. Forest plot analyses of specificities with Tsoi et al. excluded. Primary cytoreductive cases (**A**) separated from secondary cytoreductive cases (**B**). Results of the individual studies are represented by the circles. Circle sizes represent the weight of the studies. Pooled specificities are shown by the squares. Horizontal lines indicate the confidence intervals [21–28].

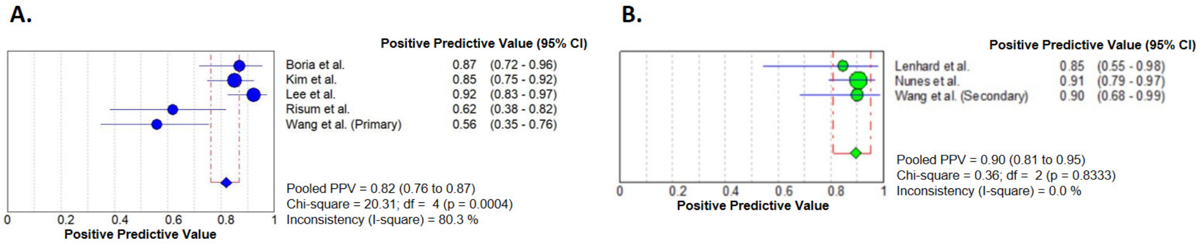

Figure S3. Forest plot analyses of positive predictive values with Tsoi et al. excluded. Primary cytoreductive cases (**A**) separated from secondary cytoreductive cases (**B**). Results of the individual studies are represented by the circles. Circle sizes represent the weight of the studies. Pooled PPVs are shown by the squares. Horizontal lines indicate the confidence intervals [21–28].

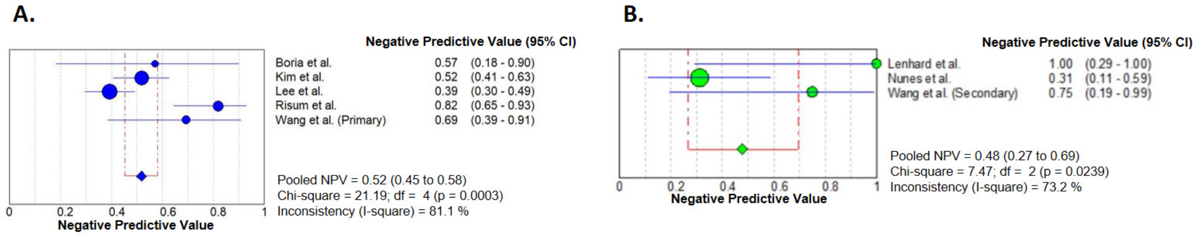

Figure S4. Forest plot analyses of negative predictive values with Tsoi et al. excluded. Primary cytoreductive cases (**A**) separated from secondary cytoreductive cases (**B**). Results of the individual studies are represented by the circles. Circle sizes represent the weight of the studies. Pooled NPVs are shown by the squares. Horizontal lines indicate the confidence intervals [21–28].

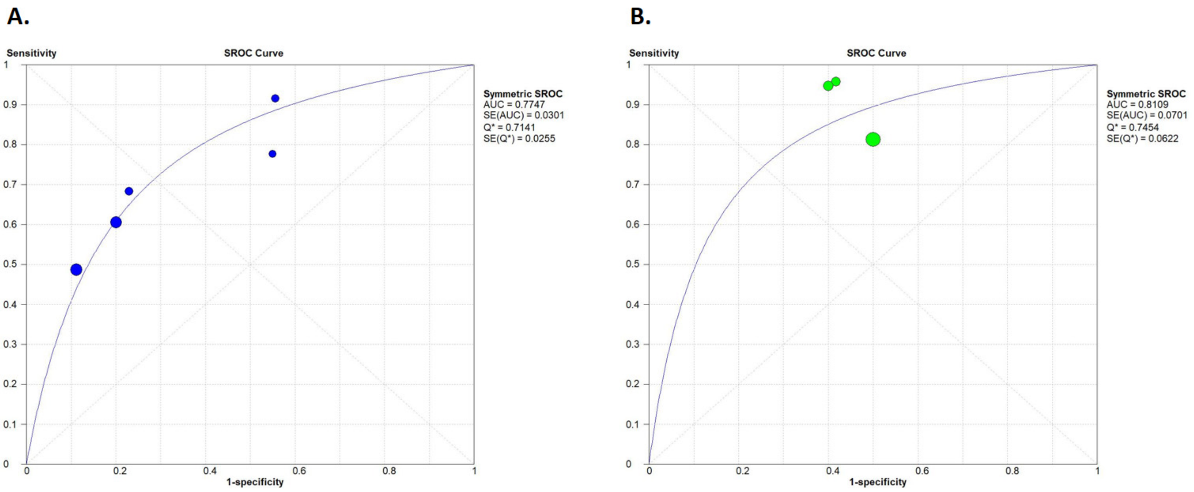

Figure S5. Summary receiver operating characteristic (SROC) curves with Tsoi et al. excluded. Primary cytoreductive cases (**A**) separated from secondary cytoreductive cases (**B**). Results of the individual studies are represented by the circles. Circle sizes represent the weight of the studies. Q* indicates the point in which the value of sensitivity equals to the value of specificity [21–28].
